# Development of a protocol for fractionating and characterising fibres from lignocellulosic food waste

**DOI:** 10.1016/j.fochx.2024.101501

**Published:** 2024-05-24

**Authors:** Clara Pedrazzani, Pio Viscusi, Andrea Fuso, Augusta Caligiani

**Affiliations:** Department of Food and Drug, University of Parma, Parco Area delle Scienze 17/A, 43124 Parma, Italy, Parma 43124, Italy

**Keywords:** Dietary fibre, Food by-products, Fractionation, Soluble fibre, Insoluble fibre, Hemicellulose (PubChem SID: 405236826), Cellulose (PubChem CID: 16211032), Pectin (PubChem SID: 482024439), Lignin (PubChem CID: 175586).

## Abstract

This study aims to explore an advanced protocol for characterising dietary fibre (DF) fractions to meet the growing demand for accurate and reliable data. Although current enzymatic-gravimetric approaches, e.g., AOAC and Van Soest analysis, provide information about soluble and insoluble DF quantification, they present limitations related to the lack of fractions characterisation. To overcome these limitations, the proposed protocol integrates the official AOAC 991.43 method with the sequential fibre fractionation by exploiting the different resistance of the fibre fractions to acid hydrolysis treatments (TFA and H_2_SO_4_), utilising hazelnut shells as a case-study. Each hydrolysed fraction was quantified and characterised through GC–MS analysis of monosaccharides. The data obtained for hemicellulose, cellulose, and lignin fractions were then discussed and compared with the Van Soest method. This approach yields a comprehensive procedure applicable to different food and nutraceutical products, emphasising the importance of DF characterisation for a deeper understanding of their bio-functional properties.

## Highlights

• A protocol for dietary fibre fractionation and quantification was developed.
• Fibre was fractionated by exploiting the resistance to acid hydrolysis treatments.
• Fibre fractions were quali- and quantified in terms of monosaccharides by GC–MS.
• The developed method was compared with gravimetric and other chemical methods.
• Advanced characterisation techniques are essential for enhancing knowledge of DF.

## Graphical abstract

**Figure:**
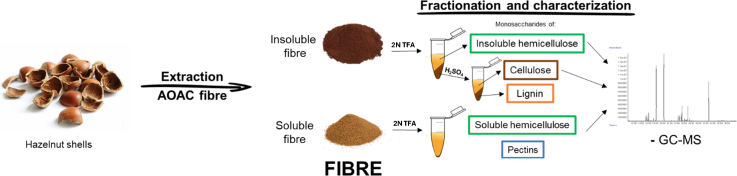

## 1 Introduction

Dietary fibre (DF) has been widely studied for its functional and beneficial properties, making it a crucial component of a healthy diet (Dhingra, Michael, Rajput, & Patil, 2012). Its importance mainly relates to its ability to promote digestive health, regulate blood sugar levels, and contribute to weight management (Barber, Kabisch, Pfeiffer, & Weickert, 2020). Furthermore, in recent years, there has been a growing interest in exploring the potential of food by-products rich in different types of fibres. The valorisation of food waste represents a significant step towards a more sustainable circular economy (Fuso et al., 2021). However, the enlargement of the portfolio of fibre ingredients and advances in nutritional studies have not been accompanied by comparable progress in developing reliable and comprehensive methods for the detailed characterisation of these molecules (Alyassin & Campbell, 2019). Knowledge of the composition of different DF fractions is fundamental to understanding the structure-activity relationship and explaining their functionality and potential exploitation in food and nutraceuticals. For this purpose, it is also important to have a method of high applicability. It is well known that the health-promoting effects of DFs are structure-dependent. Thus, by providing detailed information on the types of fibre found in foods, it would be possible for the researchers to identify fibres that contribute to specific health benefits (Tunçil, 2020). In addition to the beneficial effects, DF is exploited for its chemical and physical properties (e.g. solubility, ability to bind fats, ability to retain water, density, swelling capacity, and ability to form gels). For this reason, they are utilised incorporating them into various food products. To achieve this, it is necessary to extract the fibre of interest and eliminate the unwanted compounds. The selection of the extraction method used to isolate fibres strongly depends on the composition of the target fibre, its complexity, chemical nature, degree of polymerisation, and the presence of oligosaccharides. This emphasises the importance of having a comprehensive characterisation of the source material for both industrial and research applications (Maphosa & Jideani, 2016).

There are several types of fibre with different functions. DF is classified as soluble and insoluble based on its solubility in water. Specifically, soluble dietary fibre (SDF) includes pectin, inulin, β-glucans, and other polysaccharides, while insoluble dietary fibre (IDF) comprises cellulose, hemicelluloses, lignin, and resistant starch (Ma, Zhang, Zhai, & Tan, 2023).

Currently, DF is determined using the AOAC official enzymatic-gravimetric methods (e.g. AOAC Official method 991.43, 1995 and subsequent modifications), which primarily separate SDF and IDF fibre without giving information on the distribution of soluble and insoluble fibre fractions. This issue has been addressed by another widespread gravimetric method, the Van Soest analysis, which divides the IDF into neutral and acid detergent fibre enabling the gravimetric quantification of hemicellulose, cellulose, and lignin (Van Soest, Robertson, & Lewis, 1991). However, the measurements based on gravimetric determinations may result in ‘gross errors’, mainly due to the co-presence of other components in the fibre fraction that are not properly considered, especially when the methods deal with a wide variety of fibre-rich sources. Moreover, it has been shown that although different methods may produce comparable DF values, the composition can vary considerably (Wolters, Verbeek, Van Westerop, Hermus, & Voragen, 1992). This issue highlights the importance of employing methods capable of precisely determining the components of DF from different matrices.

Many approaches have been proposed for an in-depth characterisation of DF, based on advanced analytical techniques such as HPLC-MS, GC–MS, or NMR (Alyassin et al., 2019). To precisely define the structure of a DF, a combination of techniques is required, depending on the desired level of detail, embracing molecular weight (MW) determination, type of glycosidic linkage, presence of ramifications or decorations in the main polysaccharide backbone, and monosaccharide composition (Bauer, 2012). Consequently, the full characterisation of a DF is a time-consuming approach that is not feasible for routine application. One of the simplest and most informative analysis is the composition of monosaccharides, deriving from the complete hydrolysis of the polysaccharide chain. While this method results in the loss of information about sequence and glycosidic bond type, advanced techniques such as NMR spectroscopy and mass spectrometry are employed to precisely determine these aspects. Despite this limitation, the analysis of monosaccharides is generally recognised as one of the most important in DF characterisation thanks to the possibility of inferring, from monosaccharide distribution, the type of polysaccharides present in a complex DF (Garcia-Vaquero, 2019). Moreover, common analytical techniques for monosaccharide composition, such as GC–MS or HPLC-MS methods, are fully quantitative and enable accurate estimation of the total fibre content of the analysed fraction (Alyassin et al., 2019). Polysaccharides can be hydrolysed to monosaccharides mainly in the presence of concentrated sulphuric acid (H_2_SO_4_), which leads to the complete hydrolysis of polysaccharides, giving lignin as solid residue (Melton & Smith, 2001). However, this method does not allow for distinction between different fibre types (e.g. soluble fibre, hemicellulose, and cellulose) as it is non-selective hydrolysis and has some unavoidable side reactions, causing mass losses due to the formation of degradation products (Mosier, Ladisch, & Ladisch, 2002). Alternatively, polysaccharides can be hydrolysed in the presence of trifluoroacetic acid (TFA), which offers milder treatment and lower losses (Melton et al., 2001; Fuso et al., 2022). However, it is less effective than H_2_SO_4_ and can hydrolyse soluble fibre and hemicellulose but does not allow for the hydrolysis of cellulose. Due to the limitations of both H_2_SO_4_ and TFA hydrolysis methods, a mixed approach could achieve sequential and selective hydrolysis. This combination may overcome the individual disadvantages, allowing for the distinction between different fibre types while minimising mass losses and side reactions.

This study proposes a protocol for the qualitative and quantitative determination of fibre fractions, implementing the official gravimetric methods to improve knowledge on the starting material for its subsequent use in research and commercial applications. Hazelnut shells were selected as a case-study matrix due to their widespread availability and potential as a fibre rich food by-product. SDF and IDF were obtained following the AOAC method, and sequentially fractionated by exploiting the different resistance of the fibre fractions to acid hydrolysis treatments. Each individual fraction (soluble fibre, hemicellulose, cellulose, and lignin) was subsequently characterised. The quantitative data obtained for the IDF fractions were then compared with those obtained using Van Soest method. The limitations of the gravimetric method have been discussed, and the new approach resulted in a more accurate protocol.

## 2 Materials and methods

### 2.1 Chemicals and reagents

Alpha-amylase, amyloglucosidase from *Aspergillus niger*, ammonium hydroxide solution (NH_4_OH ACS reagent, 28.0–30.0% NH_3_ basis), barium hydroxide (Ba(OH)_2_), cellulose powder, cetyltrimethylammonium bromide technical grade, d-glucose, d-fructose, D-arabinose, D-galactose, D-mannose, D-rhamnose, d-ribose, D-xylose, D-fucose, D-galacturonic acid, D-glucuronic acid, disodium ethylenediaminetetraacetate, disodium phosphate anhydrous, 2-ethoxyethanol, Kjeldahl defoamers, *N*,*O*-Bis(trimethylsilyl)trifluoroacetamide (BSTFA), 2-(N-morpholino)ethanesulfonic acid (MES), N-octanol, phenyl β-d-glucopyranoside, protease from *Bacillus licheniformis,* sodium lauryl sulfate, trifluoroacetic acid (TFA), tris(hydroxymethyl)aminomethane (TRIS), sodium borate decahydrate, sodium nitrate (NaNO3), sulphuric acid (96% H_2_SO4), ultrapure water obtained with Milli-Q® system were purchased from Merck (Merk, Darmstadt, Germany). Diethyl ether, dimethylformamide (DMF), and ethanol (EtOH) were purchased from Carlo Erba reagents (Carlo Erba, Milan, Italy). Methanol (MeOH) was purchased from VWR International (VWR International, Milan, Italy). Standard pullulans were purchased from Agilent Technologies (Agilent Technologies, Santa Clara, CA, USA).

### 2.2 Hazelnut shell samples

Hazelnut (*Corylus avellana L.*) shells were provided by a food company. The shells were ground using the IKA® A11 basic analytical mill (IKA®-Werke GmbH & Co, Staufen, FR, Germany) and sieved with a laboratory vibratory sieve shaker (Giuliani Tecnologie srl, Torino, Italy) to recover the fraction with particle size <500 μm. The samples were stored at −20 °C until the beginning of the analyses.

### 2.3 Proximate composition analysis

Proximate composition was conducted on hazelnut shells. Moisture, dietary fibre, lipid, protein, and ash contents were determined using standard procedures (AOAC, 2002). Moisture was determined in oven at 105 °C for 24 h. Insoluble and soluble fibre content was quantified by the AOAC 991.43 official enzymatic-gravimetric method for DF. Crude lipid content was determined using an automatised Soxhlet extractor (SER 148/3 VELP SCIENTIFICA, Usmate Velate, Italy) using diethyl ether. Proteins were determined with a Kjeldahl system (DKL heating digestor and UDK 139 semiautomatic distillation unit; Velp Scientifica, Usmate Velate, Italy) by using 6.25 as nitrogen-to-protein conversion factor. Total ash was determined after mineralisation in the muffle furnace Ionos 201 (Mazzali Fratelli, Monza, Italy) at 550 °C for 5 h.

### 2.4 Methods for dietary fibre fractionation and monosaccharide characterisation

#### 2.4.1 Extractions of IDF and SDF

The extraction of IDF and SDF fractions from hazelnut shells followed the AOAC procedure, with slight modifications (AOAC 991.43). Briefly, 1 g of ground hazelnut shells underwent sequential enzymatic digestion. Initially, 50 μL of amylase was added and incubated at 95–100 °C for 15 min, followed by the addition of 100 μL of protease at 60 °C for 30 min, and finally, 300 μL of amyloglucosidase at 60 °C for 30 min at a pH of 4.0–4.7, to remove starch and proteins. The hydrolysed samples containing IDF and SDF were washed with 20 mL of hot water to solubilise the SDF fraction. Subsequently, the samples were centrifuged for 15 min at 3197 ×g *(5810R Eppendorf, Hamburg, Germany).* The solid residue, collected as IDF, was separated from the supernatant containing SDF. The SDF was precipitated by adding four volumes of ethanol and recovered after another centrifugation step for 15 min at 3197 ×g. Both fractions were dried at 40 °C for 24 h and stored for further treatments. The residual protein and mineral content were calculated on both fractions by Kjeldahl and mineralisation analyses, respectively.

#### 2.4.2 Hydrolysis with trifluoroacetic acid

For SDF and IDF, obtained as described in the previous paragraph (2.4.1), 25 mg was weighed into Pyrex glass tubes and mixed directly with 3 mL of 2 mol/L TFA, to hydrolase most of the fibre. The reaction was set at 110 °C for 2 h in an oil bath with constant agitation, following the method described by Fuso et al. (2022). Under these conditions, SDF was completely hydrolysed, allowing for the analysis of its monosaccharide composition, whereas IDF was partially solubilised. More precisely, only hemicellulose was hydrolysed, while cellulose and lignin remained in the pellet. The samples were centrifuged for 15 min at 3197 ×g. The supernatant, containing the monosaccharides resulting from the SDF and IDF fraction, was stored for the GC–MS analysis. The IDF pellet, containing cellulose and lignin, was dried in oven at 40 °C overnight and stored for further treatments.

Therefore, after the first hydrolysis step, it was possible to know the monosaccharide composition of SDF and hemicellulose from IDF. Furthermore, the sum of these monosaccharides allowed to obtain the total amount of these fibre fractions.

#### 2.4.3 Hydrolysis with sulphuric acid

The residual pellet of IDF obtained from the first step of hydrolysis with TFA was subjected to a second step of hydrolysis with H_2_SO_4_, aiming at hydrolysing cellulose. Three different hydrolysis methods were tested on standard cellulose, as no official method exists.

##### 2.4.3.1 First method of hydrolysis with sulphuric acid

The Van Soest condition was applied with some modifications (Van Soest et al., 1991). The reaction system consisted of the residual IDF and 0.5 mL of 12 mol/L H_2_SO_4_ in Pyrex glass tubes. The reaction was carried out in a water bath at 20–23 °C for 3 h with constant agitation.

##### 2.4.3.2 Second method of hydrolysis with sulphuric acid

The protocol described by Ma (Ma, Zhao, Zhang, & Wang, 2011) was followed with some modifications. The residual IDF sample was added to 0.5 mL of 12 mol/L H_2_SO_4_ in Pyrex glass tubes and left to react in a water bath for 1 h at 50 °C with constant agitation.

##### 2.4.3.3 Third method of hydrolysis with sulphuric acid

The protocol described by Miura (Miura, Lee, Inoue, & Endo, 2012) was followed with some modifications. The residual IDF sample was added to 0.5 mL of 12 mol/L H_2_SO_4_ in Pyrex glass tubes and left to react in a water bath for 1 h at 50 °C with constant agitation. The acid was then diluted by adding 3 mL hot distilled water to obtain 2 mol/L H_2_SO_4_ and left to react for 1 h at 100 °C with constant agitation (Mikame & Funaoka, 2006; Miura et al., 2012).

After each hydrolysis method, the H_2_SO_4_ was diluted with hot distilled water to 0.1 mol/L. The reaction mixture was centrifuged at 3197 ×g for 10 min. The pellet was kept for lignin quantification. The solution was then neutralised using 0.1 mol/L Ba(OH)_2_ to remove H_2_SO_4_ in the form of barium sulphate, and centrifuged. The supernatant, containing cellulose-derived monosaccharides, was kept for the GC–MS analysis.

#### 2.4.4 Characterisation of each fraction in terms of monosaccharide composition

The monosaccharide composition of SDF and IDF fractions in hazelnut shells was determined by gas chromatographic analysis combined with mass spectrometry (GC–MS), following the protocol described by Fuso et al. (2022) with some modification. Briefly, 850 μL of each solution and 150 μL of 1 mg/mL phenyl β-D-glucopyranoside, used as internal standard, were evaporated using a rotavapor R-215 (BÜCHI, Flawil, SG, Switzerland). The resulting dried hydrolysate was washed with 1 mL of methanol to remove potential TFA residue and evaporated again. 1 mL of 0.5 mol/L NH_4_OH was subsequently added to delactonise any eventually present sugar acid lactones in the solution, which do not derivatise leading to an underestimation of uronic acids, and again evaporated. The dried hydrolysate was dissolved in 800 μL of dimethylformamide (DMF) and 200 μL of *N*,*O*-Bis(trimethylsilyl)trifluoroacetamide (BSTFA), used as derivatising agent. The reaction was held for 1 h at 60 °C. GC–MS analysis of monosaccharides was performed with a 6890 N gas chromatograph coupled to a 5973 N mass selective detector (Agilent Technologies, Santa Clara, CA, USA). An SLB-5 ms, 30 m × 0.25 mm, 0.25 μm of thickness column (Supelco, Bellefonte, PA, USA) was used. The chromatogram was acquired utilising the scan mode (*m/z* 40–500), with programmed temperature ranging from 60 °C to 270 °C. The initial temperature was set to 60 °C and held for 2 min, followed by a linear ramp to 160 °C at a rate of 10 °C/min. Subsequently, an isothermal dwell of 5 min was maintained, succeeded by a further ramp to 220 °C at the same rate, and held for an additional 5 min. Final temperature was increased to 270 °C at a rate of 20 °C/min, and maintained for 5 min.

Quantification of monosaccharides in SDF and hemicellulose from IDF was performed using response factors, considering the area and concentration ratios between the internal standard (phenyl β-D-glucopyranoside) and various monosaccharides (d-glucose, d-fructose, D-arabinose, D-galactose, D-mannose, D-rhamnose, d-ribose, D-xylose, D-fucose, D-galacturonic acid, D-glucuronic acid).

Glucose quantification of cellulose from IDF was performed with response factors, considering the area and concentration ratios between the internal standard (phenyl β-D-glucopyranoside) and glucose. Furthermore, pure cellulose was used as an external reference standard. Cellulose was hydrolysed under the strong sulphuric acid conditions, releasing glucose. However, since the degradation of the monomer also occurs, it must be considered in the final quantification of the cellulose in the sample. Pure cellulose was subjected to the same hydrolysis conditions as the samples (Paragraph 2.4.3.3). From the glucose obtained after the hydrolysis, the degradation rate was calculated as follows:

% degradation rate: 100 – (glucose amount/pure cellulose amount * 100).

This value was then used to correct the amount of glucose obtained in the samples.

#### 2.4.5 Quantification of lignin

Lignin was quantified by subtracting the amount of hemicellulose and cellulose, which are calculated from GC–MS analysis, from the initial weight of the IDF, as follows:

Lignin = IDF – (hemicellulose + cellulose).

The subtraction method was chosen instead of using the residual weight because, during the acid hydrolysis of IDF, part of the lignin might solubilise, potentially leading to an underestimation of the residual pellet (Godin, Agneessens, Gerin, & Delcarte, 2014).

### 2.5 Soluble fibre analysis by HPSEC-RID

The SDF was analysed by high-pressure size-exclusion chromatography (HPSEC-RID) to determine the MW of the SDF constituent molecules (Table S1). For the analysis, an Agilent 1260 Infinity II LC system equipped with a refractive index detector (RID) was used (Agilent Technologies, Santa Clara, CA, USA). The compounds were separated by employing a 20.8 μ, 7.5 mm × 300 mm PL aquagel-OH MIXED-M column (Agilent Technologies, Santa Clara, CA, USA). 0.1 mol/L NaNO_3_in water was used as eluent for the analysis, with a flow rate of 0.7 mL/min. The column and RID temperatures were 30 and 35 °C, respectively. The calibration of the instrument was performed using standard pullulans at different MW: 9.8 Kg/mol; 22 Kg/mol; 49.4 Kg/mol; 106 Kg/mol; 201 Kg/mol. The standards were prepared at a final concentration of 1 mg/mL in NaNO_3_ solution. After calibration with the standards, duplicate analysis was conducted on the SDF samples. Specifically, 5 mg of dry sample was weighed and dissolved in 5 mL of NaNO_3_ solution. The samples were placed in a water bath at 35 °C for approximately 1 h to promote solubilisation before injection.

### 2.6 Van Soest gravimetric method

The Van Soest method was conducted to compare our protocol with an existing method for quantifying different insoluble fibre fractions (hemicellulose, cellulose, and lignin) (Van Soest et al., 1991). According to the principle of the detergent fibre analysis, plant cells can be divided into less digestible cell walls, containing hemicellulose, cellulose, and lignin and mostly digestible cell contents containing starch and sugars. Van Soest separated these two components using a neutral detergent solution (ND) containing sodium-lauryl sulfate and EDTA, allowing the recovery of a fibre residue composed of the major cell wall components: lignin, cellulose, and hemicellulose, and an acid detergent solution (AD) containing 1 mol/L H_2_SO_4_, allowing the recovery of cellulose and lignin. Briefly, for NDF determination, 1 g of ground sample was mixed with 100 mL of ND solution and heated at 100 °C under reflux for 1 h. The solution was then filtered, and the pellet was washed three times with boiling water and then twice with cold acetone. The NDF was dried for 8 h at 105 °C. The residual minerals were then analysed in a muffle furnace at 550 °C for 2 h. The NSF is calculated as follows:

$$\mathrm{NDF}\%=\left(\left(g\;\text{of residue}–g\;\text{of}\;\mathrm{ash}\right)/g\;\text{of sample}\right)\;x\;100.$$

where NDF = Hemicellulose + Cellulose + Lignin.

For ADF determination, 1 g of ground sample was mixed with 100 mL of AD solution and heated at 100 °C under reflux for 1 h. The solution was filtered, washed three times with boiling water and then twice with cold acetone. The ADF was dried for 8 h at 105 °C. The residual minerals were then analysed in a muffle furnace at 550 °C for 2 h. The ADF is calculated as follows:

$$\mathrm{ADF}\%=\left(\left(g\;\text{of residue}–g\;\text{of}\;\mathrm{ash}\right)/g\;\text{of sample}\right)\;x\;100.$$

where ADF = Cellulose + Lignin; Hemicellulose = NDF – ADF.

To quantify lignin, the fibre was added to 72% H_2_SO_4_ and heated at 20–23 °C for 3 h. The resulting pellet was filtered and washed with hot water until pH neutrality. Lignin was dried for 8 h at 105 °C. The residual minerals were then analysed in a muffle furnace at 550 °C for 2 h. Lignin and cellulose were quantified as follows:

$$\text{Lignin}\%=\left(\left(g\;\text{of residue}–g\;\text{of}\;\mathrm{ash}\right)/g\;\text{of sample}\right)\;x\;100.$$

Cellulose%=ADF–lignin.

### 2.7 Statistical analysis

All statistical analyses were performed using IBM SPSS v.23.0 software (SPSS Italia, Bologna, Italy). Experimental data are expressed as the mean of three replicates ± standard deviation. The data were subjected to a *t*-test to assess differences among the samples. Significant differences were compared at the level of *p* < 0.05. Method was validated in terms of accuracy (closeness of a measured value to its true value) and precision (closeness of repeated measurements to the same item) (ICH Harmonized Tripartite Guideline, 2005 Validation of analytical procedures: text and methodology. Q2 (R1) 1.20:05).

## 3 Results and discussion

### 3.1 Quantification of hazelnut shell fibre and other components through official methods

Hazelnut shells were first analysed for their proximate composition, according to the official methods described in section 2.3. The analysis was performed in triplicate and the results are expressed as a percentage of dry matter (g/100 g DM) (Table 1). The moisture content was 7.1 ± 0.1%. Fibre represented the main and almost the whole constituent of hazelnut shells with a percentage of 89.3 ± 0.1%. This high fibre content confirmed hazelnut shell a valid case-study for the development of a new quali-quantitative protocol for fibre characterisation. All the other components, i.e. lipids, proteins, and ash, were below 3%, representing a minimal portion of this biomass.

**Table 1:** Proximate composition of hazelnut shells expressed as g/100 g DM^a^.

**Parameter**	**Hazelnut shells (g/100 g DM)**	**Method**
Total protein	2.2 ± 0.1	Kjeldahl
Crude lipid	3.0 ± 0.9	Soxhlet (diethyl ether)
Ash	2.1 ± 0.1	Oven, 550 °C, 5 h
		
Soluble fibre (SDF)	3.3 ± 0.1^⁎^	AOAC, 991.43
Insoluble Fibre (IDF)	86.0 ± 0.1^⁎^	
		
Insoluble Fibre (NDF)	89.9 ± 0.5^⁎^	Van Soest
Hemicellulose	24.7 ± 0.4^⁎^	
Cellulose	23.8 ± 0.3	
Lignin	41.5 ± 0.7	
		
Soluble fibre (SDF)	2.6 ± 0.2^⁎^	Protocol developed
Hemicellulose + pectin	19.8 ± 0.6*	
Cellulose	24.3 ± 0.3	
Lignin	42.0 ± 1.0	

a Results represent the mean ± SD of three independent analyses. Means with * are significantly different (*p < 0.05*) according to *t*-test.

Fibres can be subdivided, according to their solubility, into a water soluble (SDF) and insoluble (IDF) component. The analyses conducted to measure these two components using the official method (AOAC, 991.43) revealed, as expected, a predominance of IDF (86.0 ± 0.1%) compared to SDF (3.3 ± 0.1%), in accordance with a previous study (Fuso et al., 2023). Insoluble fibre fractions were further characterised by applying the Van Soest method (Van Soest et al., 1991). Using this method, the IDF content, expressed as the sum of hemicellulose, cellulose, and lignin (NDF), was 89.9%, slightly higher (*p* < 0.05) than that obtained by the AOAC method. This difference may be due to the neutral detergent solution used in the Van Soest method, which dissolves non-fibrous residues and a portion of the soluble fibre. Consequently, a fraction of the soluble fibre is quantified within the pellet (NDF), resulting in an IDF overestimation compared to that quantified with the AOAC method (Alyassin et al., 2019). Furthermore, this can also be explained by the fact that the NDF residue contains some proteins and pectin when the analysed biomass has a high content of these components (Godin et al., 2014).

The quantitative data obtained from the official methods of analysis were then compared with those obtained from the protocol developed, in the following paragraphs.

### 3.2 Development of a protocol for simultaneous quantification and characterisation of dietary fibre

**Fig. 1:**
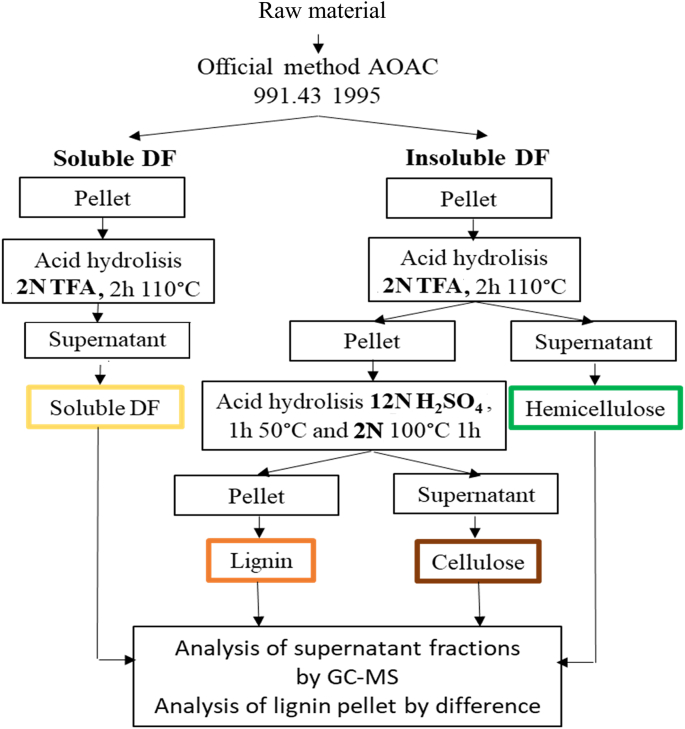
Flow diagram of the experimental protocol for the fractionation of dietary fibre.

The experimental protocol proposed for fibre fractionation is schematised in the flowchart shown in Fig. 1.

**Fig. 2:**
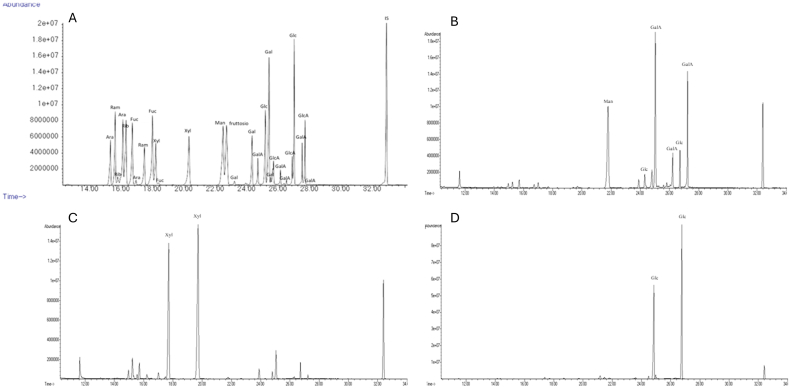
GC–MS chromatograms of monosaccharide standards (A) and monosaccharides from TFA hydrolysis of SDF (B), TFA hydrolysis of IDF (C), and H_2_SO_4_ hydrolysis of IDF (D) obtained from hazelnut shells.

The proposed protocol starts from the SDF and IDF pellet obtained through the enzymatic-gravimetric AOAC method and exploits the different resistance of the fibre fractions that constitute SDF and IDF fibre to different acid hydrolysis treatments with TFA and H_2_SO_4_. In contrast to conventional gravimetric methods, like the Van Soest's, which quantify the residual pellet, our method focuses on quantifying the compounds present in the supernatant. This approach allows for a more targeted analysis. Specifically, each fibre fraction undergoes selective hydrolysis and is broken down into its constituent monosaccharides. The sum of the latter allows the quantification of the specific fibre fraction. Utilising the GC–MS analytical method, it is possible to effectively separate and identify all monosaccharides, as shown in Fig. 2 (Xia, Wang, Sun, Liang, & Kuang, 2018).

This approach offers several advantages over traditional methods. Focusing on the supernatant, it provides a more accurate reflection of fibre composition. Moreover, using GC–MS enhances specificity and sensitivity in monosaccharide detection, enabling a more comprehensive analysis of fibre fractions.

#### 3.2.1 Soluble fibre (SDF) quantification and composition

The soluble fibre obtained from the AOAC 991.43 procedure was hydrolysed in 2 mol/L TFA. The SDF should be completely hydrolysed in those conditions (Wikiera, Mika, Starzyńska-Janiszewska & Stodolak, 2015) allowing for the analysis of its constituent monosaccharides and the SDF total amount determination.

The prevailing monosaccharide in the SDF of hazelnut shells was galacturonic acid (71.7 ± 5.9%), followed by mannose (21.5 ± 4.6%), glucose (3.0 ± 0.6%), arabinose (1.1 ± 0.2%), rhamnose (1.1 ± 0.1%), and galactose (0.7 ± 0.1%), as shown in Table 2. The other monosaccharides were <0.5%.

**Table 2:** Monosaccharide composition of SDF and IDF of hazelnut shells. Results are reported as mean ± standard deviation of three independent analyses and expressed as g/100 g of total monosaccharides.

	SDF (2 mol/L TFA)	IDF (2 mol/L TFA) (Hemicellulose)	IDF (12 mol/L H_2_SO_4_) (Cellulose)
**Monosaccharide**	**g/100 g monosaccharides**		
Arabinose	1.1 ± 0.2	4.6 ± 0.8	–
Rhamnose	1.1 ± 0.1	5.5 ± 0.6	–
Ribose	0.17 ± 0.02	–	–
Fucose	0.11 ± 0.02	–	–
Xylose	0.5 ± 0.2	78.3 ± 0.8	8.3 ± 0.6
Mannose	21.5 ± 4.6	0.9 ± 0.4	1.0 ± 0.6
Fructose	–	–	–
Galactose	0.7 ± 0.1	5.2 ± 0.8	
Glucose	3.0 ± 0.6	2.4 ± 0.1	89.8 ± 1.9
Galacturonic acid	71.7 ± 5.9	3.2 ± 1.0	–
Glucuronic acid	–	–	–

The availability of monosaccharide composition, besides allowing a specific quantification of carbohydrate content, provides simultaneous information about the type of soluble fibre present in the sample (Tunçil, 2020). The net prevalence of galacturonic acid in hazelnut shell SDF suggests the presence of pectin. Furthermore, the presence of small amount of arabinose, galactose, xylose, and rhamnose reflects the neutral sugar's distribution characteristic of pectin (Fuso et al., 2023; Mohnen, 2008), confirming that pectin is the main component of the SDF in hazelnut shells.

Furthermore, the presence of mannose (21.5 ± 4.6%) and glucose (3.0 ± 0.6) suggests the presence of non-pectic polysaccharides as well, possibly glucomannans or galactoglucomannans (considering the presence of galactose). The presence of galactoglucomannans in the SDF of hazelnuts was previously reported by Tunçil, 2020, supporting the obtained data.

The presence of two main classes of polysaccharides in SDF was also confirmed by size-exclusion chromatography (Table S1), highlighting a prevalence (80%) of a polymer with MW > 41.8 Kg/mol and a smaller portion of polysaccharides with MW lower than 6 Kg/mol.

#### 3.2.2 Insoluble dietary fibre quantification and composition

The IDF obtained from hazelnut shells following the AOAC 991.43 protocol was fractionated into hemicellulose, cellulose, and lignin by exploiting the different resistance of the fractions to different acid hydrolysis treatments followed by the analysis of their constituent monosaccharides (Table 1). The IDF residue was sequentially treated with 2 mol/L TFA and 12 mol/L H_2_SO_4_ to achieve this fractionation.

The results obtained using this procedure were compared with those obtained through the Van Soest method (1991) in the paragraph 3.2.3, with the aim of comparing a chemical method with a gravimetric one.

##### 3.2.2.1 Hemicellulose quantification and characterisation

The IDF obtained by following the AOAC method was first treated with 2 mol/L TFA to hydrolyse the hemicellulose portion. TFA hydrolysis is known to result in a significantly higher xylose yield due to more efficient depolymerisation of hemicellulose compared to other acids (Guo, Zhang, Ha, Jin & Morgenroth, 2012).

The main monosaccharide observed in the IDF after the hydrolysis with 2 mol/L TFA was xylose (78.3 ± 0.8%), followed by rhamnose (5.5 ± 0.6%), galactose (5.2 ± 0.8%), arabinose (4.6 ± 0.8%), galacturonic acid (3.2 ± 1.0%), glucose (2.4 ± 0.1%), and mannose (0.9 ± 0.4%) as shown in Table 2.

The high presence of xylose suggested the effective solubilisation of xylan-rich hemicellulose with this treatment, which is consistent considering the findings from a previous study indicating hazelnut shells contain a high amount of xylan (Surek & Buyukkileci, 2017). Furthermore, the presence of arabinose could suggest its binding to the xylan backbone, as xylan in hazelnut shell was found to be substituted with small quantities of arabinans (Fuso et al., 2023).

The simultaneous presence of rhamnose, galactose, and galacturonic acid could suggest that this process led to the *co*-depolymerisation and thus solubilisation of pectic polysaccharides, such as rhamnogalacturonan, arabinan, and arabinogalactan, as proposed by Tunçil (2020). Thus, most of the pectin was found in the soluble fraction, while a more resistant part remained in the IDF portion.

Finally, the presence of small amount of glucose, mannose, and galactose could suggest the presence of galacto-glucomannans, consistent with their identification in the hemicellulose fraction of pine nut shells (Queirós et al., 2020).

##### 3.2.2.2 Cellulose quantification and characterisation

Cellulose has a strong structural resistance and requires severe hydrolysis conditions, such as the use of concentrated H_2_SO_4_, to break its glycosidic bonds effectively (Wyman, Decker, Viikari, Brady, & Himmel, 2005). The need for harsh hydrolysis conditions leads to the formation of degradation products (Mosier et al., 2002), representing a key factor to be considered when quantifying cellulose-derived monosaccharides by GC–MS. Cellulose hydrolysis is consequently more challenging and there are few unstandardised methodologies compared to hemicellulose, which is generally treated with TFA as a standardised method (Fuso et al., 2022; Melton et al., 2001; Wikiera et al., 2015).

Consequently, for the hydrolysis of the residual IDF pellet, composed of cellulose and lignin, three different methods were preliminary tested on standard cellulose to evaluate the best hydrolysis method in terms of glucose yield (Paragraph 2.4.3). The results are reported in Table 3.

**Table 3:** The percent yield of glucose obtained from cellulose standard (g/100 g of cellulose) with different H_2_SO_4_ hydrolysis conditions.

Method	Condition	Yield (g glucose /100 g of cellulose)
First method	12 mol/L H_2_SO_4_, 20–23 °C, 3 h	3.36 ± 0.02%
Second method	12 mol/L H_2_SO_4_, 50 °C, 1 h	22.9 ± 0.7%.
Third method	12 mol/L H_2_SO_4_, 50 °C, 1 h, then 12 mol/L H_2_SO_4_, 100 °C, 1 h	51.0 ± 3.0%.

The first method tested on cellulose standard (Paragraph 2.4.3.1), which adopts the conditions employed by Van Soest, thus 12 mol/L H_2_SO_4_ at 20–23 °C for 3 h, yielded only 3.36 ± 0.02% glucose from standard cellulose (Table 3). These mild conditions were probably sufficient to break down cellulose into oligosaccharides and make it soluble, separating it from lignin for quantification according to Van Soest's method. At the same time, they were not suitable for obtaining the monomer and thus for the quantification with the proposed method. The second method (Paragraph 2.4.3.2) used a higher temperature (50 °C) for 1 h. It is reported that from 45 °C, the solubility of cellulose in concentrated H_2_SO_4_ increased and the degree of polymerisation decreased (Ioelovich, 2012). This method resulted in a glucose yield of 22.9 ± 0.7% from standard cellulose. However, the increase in temperature was not sufficient to obtain a good glucose yield. The last method (Paragraph 2.4.3.3) used stronger temperature conditions and consisted of two steps: the sample was firstly treated at 50 °C for 1 h, then the acid was diluted to 0.1 mol/L and heated at 100 °C for another 1 h. The yield in this case was 51.0 ± 3.0% from standard cellulose. It has been reported that two steps of hydrolysis are much more effective than one: a decrystallisation step leads to breaking down the crystal structure of cellulose using H_2_SO_4_ at a concentration >60% and a hydrolysis step using acid with a concentration around 20–30% lead to sugars release from the decrystallised fibre (Wen, Liao, Liu, & Chen, 2007). However, under these conditions, part of the glucose was degraded, decreasing the yield. Standard glucose was therefore hydrolysed under the same conditions, to confirm the degradation issue, giving the same yield (about 50%). For this reason, it was necessary to use, as reference for quantification, a cellulose standard subjected to the same hydrolysis conditions of the sample, allowing the correct calculation of the cellulose in the IDF sample, as described in paragraph 2.4.4.

After the optimisation of the hydrolysis conditions on standard cellulose, the IDF residual pellet (not solubilised in 2 mol/L TFA), was treated with 12 mol/L H_2_SO_4_, according to the optimised method of hydrolysis (third method). After acid treatment, the supernatant was neutralised with Ba(OH)_2_ and then prepared for the GC–MS analysis of monosaccharides.

The analysis revealed glucose as the main monosaccharide component (89.8 ± 1.9%), followed by xylose (8.3 ± 0.6%), as shown in Table 2.

The presence of xylose could be due to the TFA treatment, which did not cleave completely the hemicelluloses (Udayarajan, Mohan, & Nisha, 2022). Indeed, the plant cell wall consists of cellulose microfibrils laterally connected with hemicelluloses (Cosgrove, 2005; Gibson, 2012). The type of hemicellulose could influence the resistance of cellulose fibres. For example, the lateral groups of hemicellulose can create different types of bonds with the free -OH groups on the surface of cellulose fibrils, affecting the separation process (Khodayari, Thielemans, Hirn, Van Vuure, & Seveno, 2021). Consequently, the proposed protocol also allows to determine the portion of hemicellulose strictly linked to cellulose. Guo, Zhang, and Morgenroth (2012) confirmed the presence of an insolubilised portion of hemicellulose following the treatment with TFA. For this reason, the amount of xylose (8.3 ± 0.6) found following the treatment with H_2_SO_4_ was considered in the calculation of hemicellulose fraction (Table 2).

##### 3.2.2.3 Lignin

Following the quantification of hemicellulose and cellulose fractions through the GC–MS analysis, the lignin pellet could be quantified as the difference from the total IDF. Lignin resulted in 48.8 ± 1.1% of the total IDF.

#### 3.2.3 Comparison between the proposed protocol to gravimetric methods and current analytical methods

Gravimetric methods and selected chemical methods found in the literature are compared to the methods proposed in our protocol in order to highlight their differences, advantages, and disadvantages. Due to the absence of reference materials for fibres, accuracy was determined by comparison with independent measurements obtained through official method (Van Soest protocol and AOAC gravimetric methods). The results are reported in Table S2 and compared with accepted true values (gravimetric measurement). In the same table the precision, the bias, and the accuracy for each fibre fraction are reported as a percentage. Data precision was calculated as a % standard deviation; accuracy was determined by the following equation: accuracy = (p^2^ + b^2^)^1/2^, where p is precision and b is the bias (percentage difference between the measured value and the true or ‘accepted’ value).

The protocol compared with gravimetric methods showed that the accuracy and precision are highly acceptable for cellulose and lignin fraction (bias respect to the accepted true value 2.1 and 0.5% respectively), while for soluble fibre and hemicellulose fraction higher bias have been registered (around 20%). However, we have to take into account that the AOAC 991.43 and the Van Soest (1991) methods, being exclusively gravimetric, have limitations due to the possibility of gross errors and do not allow characterisation of the individual constituents of the soluble and insoluble fraction of DF.

According to Table 1, the SDF fraction determined with the developed protocol represents 2.6% of the proximate composition of hazelnut shells, compared to 3.3% calculated by the AOAC method. Thus, the total amount of monosaccharides in the SDF fraction accounts for 77.3% of the pellet obtained by the AOAC 991.43 method for SDF. This could indicate that the SDF was overestimated by the gravimetric method, even after the correction for the presence of proteins and minerals. It had been previously observed that the salts of the buffers, as well as other organic and inorganic components of the sample, may precipitate with the SDF (Mañas, Bravo, & Saura-Calixto, 1994), explaining the slight overestimation of SDF value observed in the gravimetric quantification. The analysis of monosaccharides in the SDF thus allows this type of error to be corrected by a more selective analysis.

The results of the IDF analysis obtained by the proposed fractionation method were compared with the data obtained by the Van Soest method (Table 1).

**Fig. 3:**
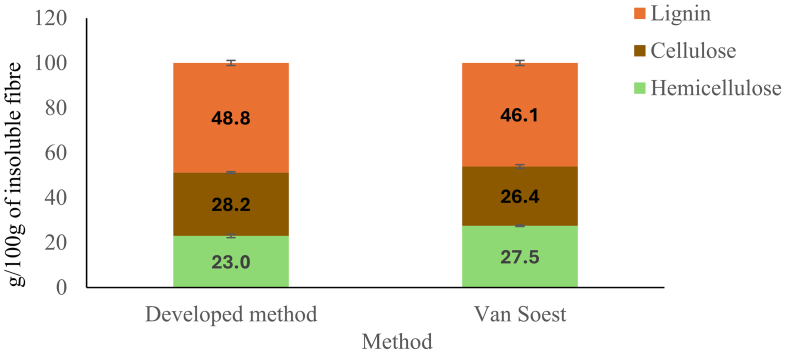
Composition of the IDF fraction of hazelnut shells according to the developed method and the Van Soest Method.

The values of hemicellulose, cellulose, and lignin in the IDF fractions obtained from the two methods are both consistent with the literature (Fig. 3). In this regard, it has been reported that hazelnut shells contain 40–51% of lignin, followed by hemicellulose with 13–32%, and cellulose with 17–29% (Fuso et al., 2021; Hoşgün, Ay, & Bozan, 2020). This result indicates that the proposed method correctly quantifies the three fractions of IDF.

Although both methods are consistent with the literature, significant differences (*p* < 0.05) were found in the hemicellulose content, which was lower when applying the new protocol (19.8%) compared to the gravimetric one (24.7%). Consequently, this resulted in a higher percentage of cellulose (24.3%) and lignin (42.0%) compared to Van Soest's method (23.8% and 41.5%, respectively). As reported by Godin (2014), the detergent fibre method shows some accuracy deficiencies. It under- or overestimates the content of cellulose and hemicellulose, and underestimates the content of lignin, depending on the sample. This variability can be explained by the fact that the NDF residue may contain some proteins, the ADF residue may contain some pectin and some insolubilised hemicelluloses, and the acid detergent lignin residue does not contain the acid soluble lignin. In agreement with our data, Godin and colleagues found a higher hemicellulose content with the gravimetric method compared to the enzymatic-chemical Uppsala method, which quantifies polysaccharides based on their monosaccharide components. This difference was observed in samples with lower levels of pectin, similarly to our case, while the opposite result was found in samples richer in pectin, indicating how the Van Soest method is extremely variable depending on the sample.

Additionally, it has been shown that although different methods can produce comparable DF values, the composition can vary considerably (Wolters et al., 1992). This highlights the importance of employing methods capable of precisely determining the well-defined components of DF. For this reason, it is important to consider additional factors, such as monosaccharide composition. Recent research has highlighted the importance of understanding the structural complexity of these components, as variations in their composition can significantly influence the properties and function of the material (Godin et al., 2014; Tunçil, 2020). On the other hand, conventional analyses like the Van Soest method may overlook such details, limiting their ability to provide comprehensive insights into the composition and characteristics of the material. Therefore, employing advanced characterisation techniques that provide a more detailed understanding of the fibre fractions is essential for enhancing our knowledge and optimising the utilisation of natural resources.

Other researchers have previously explored methods aiming to achieve greater accuracy beyond conventional gravimetric techniques. For instance, Tunçil (2020) investigated the ability of different acids to hydrolyse fibre. However, this work focused on characterising fractions in terms of monosaccharide composition rather than quantification. In contrast, our study developed a methodology enabling the simultaneous evaluation of both aspects. Furthermore, in our protocol, SDF and IDF were obtained from an established official protocol (AOAC 991.43), whereas Tunçil's approach diverged, precluding direct comparison with recognised standards for data validation.

Godin et al. (2014) also compared a method based on acid hydrolysis and subsequent monosaccharides analysis using UPLC-MS to the Van Soest method. They confirmed that the latter method was not entirely reliable, leading to an overestimation of cellulose, overestimation, or underestimation of hemicellulose, depending on the samples, and an underestimation of lignin. In contrast to our protocol, which involved sequential hydrolysis with two different acids, they performed two separate hydrolysis. One IDF sample was treated with concentrated sulphuric acid, solubilising both the hemicellulose and the cellulose, while another IDF sample was treated with dilute sulphuric acid, solubilising only the hemicellulose. By measuring the hemicellulose content in the two samples, the amount of cellulose can be calculated by difference (Godin et al., 2014). Although the approach used is similar to ours, a subtraction calculation is more susceptible to error than the sequential method. Moreover, the use of TFA presents fewer problems compared to sulphuric acid, i.e., less production of degradation products, and in addition to this, it can be easily removed due to its volatility.

These comparative analyses emphasise the importance of methodological precision in DF analysis and the need to develop a clear and standardised protocol for fibre analysis arises, in order to ensure the comparability of results obtained across multiple studies.

## 4 Conclusion

DF is increasingly recognised for its functional properties and human health benefits. However, the deep characterisation of this food component is still highly challenging, and this represent a strong limit in the accurate evaluation of its benefits. Unfortunately, this is limited by the difficulties related to the analysis of its main components, which are mainly pectin, hemicellulose, cellulose, and lignin. This new integrated protocol allows the simultaneous quantification and characterisation of these DF fractions, exploiting their different resistance to different acid hydrolysis treatments. Compared to commonly used gravimetric methods, it allows the quantification of the different fractions of the fibre and at the same time the determination of their monosaccharide composition. The ongoing development of our understanding of fibre fractions and their monosaccharides composition are key factors in exploring new paths for the characterisation of these materials and their exploitation in food and feed applications.

## Funding

This work has been carried out in the frame of the ALIFAR project, funded by the Italian Ministry of University through the program ‘Dipartimenti di Eccellenza 2023–2027.

## CRediT authorship contribution statement

**Clara Pedrazzani:** Writing – review & editing, Writing – original draft, Visualization, Validation, Software, Methodology, Investigation, Formal analysis, Data curation, Conceptualization. **Pio Viscusi:** Writing – review & editing, Software, Methodology, Investigation, Formal analysis, Data curation. **Andrea Fuso:** Writing – review & editing, Software, Methodology, Investigation, Formal analysis. **Augusta Caligiani:** Writing – review & editing, Validation, Supervision, Resources, Project administration, Funding acquisition, Conceptualization.

## Declaration of competing interest

The authors declare that they have no known competing financial interests or personal relationships that could have appeared to influence the work reported in this paper.

## Data Availability

The data that has been used is confidential.

## Appendix A Supplementary data

Supplementary material. Image 1
