# Supplementary material for: Development of a protocol for fractionating and characterising fibres from lignocellulosic food waste
Source: Food Chem X. 2024 May 24;22:101501. doi: 10.1016/j.fochx.2024.101501 (PMC11154191; doi:10.1016/j.fochx.2024.101501)
Supplement: Supplementary file 1 — Supplementary material. [file mmc1.docx]

DEVELOPMENT OF A PROTOCOL FOR FRACTIONATING AND CHARACTERISING FIBRES FROM LIGNOCELLULOSIC FOOD WASTE

Clara Pedrazzani^1^, Pio Viscusi^1^, Andrea Fuso^1^, Augusta Caligiani^1^

^1^Department of Food and Drug, University of Parma, Parco Area delle Scienze 17/A, 43124 Parma, Italy.

SDF analysis of hazelnut shells by HPSEC-RID

The HPSEC-RID was used to assess the molecular weight (MW) of the SDF extracted from hazelnut shells (Table S1).

Table S1. Molecular weight (MW), Retention time (RT), and Area % of the SDF molecules analysed by HPSEC-RID.

| **RT (min)** | **Area %** | **MW (kg/mol)** |
| --- | --- | --- |
| 10.62 ± 0.04 | 80.4 ± 1.2 | 41.8 ± 2.5 |
| 11.75 ± 0.03 | 14.4 ± 1.3 | 6.32±0.02 |
| 12.6 ± 0.1 | 2.8 ± 0.8 | 2.8 ± 0.1 |
| 13.08 ± 0.03 | 2.3 ± 0.4 | 1.64 ± 0.04 |

As shown in Table S1, the SDF fraction consisted of molecules with MW between 1.6 and 41.8 kg/mol. In particular, 80.4 ± 1.2% consisted of molecules with MW of 41.8 kg/mol, 14.4 ± 1.3% of molecules with MW of 6.3 kg/mol, 2.8 ± 0.8% of molecules with MW of 2.8 kg/mol, and 2.3 ± 0.4% of molecules with MW of 1.6 kg/mol.

Table S2. Precision (standard deviation %), bias, and accuracy of the fibre fraction quantification according to the developed protocol.

| Fibre fraction | True (accepted) value (g/100g sample^*^) | Average measured value (g/100g sample^*^, n=3) | Standard deviation % | Bias % | Accuracy |
| --- | --- | --- | --- | --- | --- |
| SDF | 3.3 | 2.6 | 7.6 | 21 | 22 |
| Hemicellulose | 24.7 | 19.8 | 3.0 | 20 | 20 |
| Cellulose | 23.8 | 24.3 | 1.2 | 2.1 | 2.4 |
| Lignin | 41.5 | 42.0 | 2.3 | 0.5 | 2.3 |

^*^g/100g sample, determined by AOAC gravimetric protocol for SDF and by Van Soest protocol for the other fraction.
